# Design and Drive Research of Nanofiber-Reinforced Polyacrylamide Hydrogels

**DOI:** 10.3390/polym18091101

**Published:** 2026-04-30

**Authors:** Kexu An, Xuewei Shi, Pengli Zhang, Hansheng Liao, Kaiming Hu, Jian Wang, Chenxing Xiang, Ning Hu

**Affiliations:** 1 School of Materials Science and Engineering, Hebei University of Technology, Tianjin 300401, China; 202321801130@stu.hebut.edu.cn (K.A.); zhangpengli_9671@163.com (P.Z.); 202331205072@stu.hebut.edu.cn (H.L.); 202331205111@stu.hebut.edu.cn (K.H.); 202331402145@stu.hebut.edu.cn (J.W.); ninghu@hebut.edu.cn (N.H.); 2 College of Aerospace Engineering, Chongqing University, Chongqing 400044, China; 3 Interdisciplinary Research Institute of Advanced Intelligent Equipment, Xihua University, Chengdu 610039, China; 4 School of Mechanical Engineering, Xihua University, Chengdu 611739, China

**Keywords:** polyacrylamide hydrogel, nanofiber reinforcement, infrared response, bilayer structure

## Abstract

Hydrogels have emerged as a crucial category of polymeric materials in materials science due to their three-dimensional network structure and remarkable capacity for water absorption and retention. However, conventional single-function hydrogels do not satisfy the increasing demands of advanced applications in biomedicine and environmental engineering. This paper focuses on the design, preparation, and performance characterization of nanofiber-reinforced polyacrylamide hydrogels to overcome this limitation. A bilayer structure, consisting of tensile layers and actuator layers based on a polyacrylamide/sodium alginate (PAM/SA) matrix integrated with functional materials, was developed. Nanocellulose (CNF) was incorporated to regulate mechanical properties by adjusting its content ratio with PAM, while poly-N-isopropylacrylamide (PNIPAM) and multi-walled carbon nanotubes (MWCNTs) were added to confer photothermal responsiveness. The deformation of the hydrogel was induced by temperature changes resulting from infrared illumination. The results indicate that the CNF-reinforced hydrogels exhibit enhanced mechanical strength—with the tensile strength reaching 17 kPa (89% higher than pure PAM) and fracture strain approaching 900% when the CNF content is 0.44 wt.% and PAM/SA mass ratio is 4:1—and they display reversible thermosensitive responses (reaching 60 °C within 100 s under near-infrared irradiation) following the incorporation of carbon nanotubes. This paper presents a novel strategy for the development of multifunctional hydrogel-based actuated systems, expanding the application potential of hydrogels in human motion tracking and drug delivery.

## 1. Introduction

Hydrogels are polymeric materials characterized by a three-dimensional network structure that are being increasingly recognized in the field of material science [1]. These materials possess distinctive physical and chemical properties [2], enabling them to absorb and retain substantial volumes of water while maintaining their physical structure [3,4,5,6], thus garnering considerable attention in numerous domains [7,8,9], especially in biomedicine and environmental protection [10]. With the development of science and technology and the rising requirements for material performance in these key fields, traditional single-function hydrogels have been gradually unable to meet the growing application demands [11,12,13,14,15,16]. For instance, in drug slow-release systems, precise control of the drug release rate is urgently needed [17]; in the sensor field, a higher sensitivity and selectivity of detection are essential [18,19,20]; in flexible electronic devices, conductive hydrogels are needed to promote effective signal transmission and enhance equipment response speed, while magnetically driven hydrogels are required to enable rapid responses to motion signals [21,22]. These practical application demands in specific scenarios render the development of multifunctional hydrogels both crucial and challenging [23,24,25,26]. Multifunctional hydrogels can overcome the limitations of single-function hydrogels and expand the application scope of hydrogel-based composites, yet achieving such multifunctionality simultaneously poses significant challenges in material design and preparation. Multifunctional hydrogels, as advanced functional materials, are gaining significant importance in both scientific research and practical applications [27,28,29]. With the development of new materials and technologies, multifunctional hydrogel will play a more important role in the future scientific and technological progress and social development.

Among various hydrogel systems, PAM/SA composite hydrogels have attracted extensive attention due to their good biocompatibility, adjustable mechanical properties, and ease of preparation. PAM provides stable covalent cross-linking networks and excellent flexibility, while SA introduces ionic cross-linking and hydrophilicity, forming a semi-interpenetrating network that synergistically optimizes the comprehensive performance of hydrogels. In recent years, significant progress has been made in the research of PAM/SA hydrogels. Researchers have improved their mechanical strength, swelling properties, and biocompatibility by adjusting polymer ratios, cross-linking methods, and introducing physical/chemical cross-linking strategies, which have been initially applied in tissue engineering scaffolds, drug delivery carriers, and water treatment materials.

Nanocellulose is a green biomass-derived nanofiller that effectively reinforces hydrogels through hydrogen bonding and physical cross-linking. Multi-walled carbon nanotubes (MWCNTs) offer excellent photothermal conversion and electrical conductivity, making them ideal for constructing light-responsive hydrogel systems [23,28]. However, most studies only employ single reinforcement or single functionality. Here, we integrate CNF reinforcement, MWCNT photothermal conversion, and PNIPAM thermal sensitivity into a bilayer PAM/SA hydrogel, thereby achieving both high mechanical strength and controllable photothermal actuation.

Multifunctional hydrogels are those that not only have excellent hygroscopicity [30,31,32] but also respond to external stimuli (e.g., temperature, light, magnetic field, etc.) or possess other specific functions (e.g., self-repairing, conductive, etc.). The design and preparation of such materials rely on the interdisciplinary integration of multiple disciplines [33,34], providing new ideas for the design of multifunctional hydrogels. By precisely controlling the synthesis conditions and post-processing methods, hydrogels can be endowed with specific structures and functions [35], facilitating the customized design of material properties. This capability for customization represents the core innovation of this paper, as it enables the tailoring of hydrogel properties to meet specific application scenarios that single-function hydrogels cannot fulfill, thereby addressing the practical demands in drug delivery and other fields. Given these advantages, multifunctional hydrogels exhibit significant application potential in areas such as human motion tracking and drug delivery systems [36,37,38,39], thereby establishing a foundation for the innovative bilayer hydrogel structure proposed in this research.

We present an innovative bilayer hydrogel structure. This structure consists of two distinct layers: tensile layers and actuator layers, each comprising a PAM/SA hydrogel matrix integrated with specific functional materials. Through manipulating the content ratio of CNF and PAM, we were able to adjust the mechanical properties of the hydrogel, rendering it suitable for diverse stretchable sensors with varying elastic moduli. Furthermore, the incorporation of PNIPAM imparts the ability to thermally stimulate responses in the hydrogels. Deformation of the hydrogel is achieved by inducing a temperature change in the hydrogel under infrared irradiation. The key factor in achieving the infrared responsiveness of composite hydrogels is the incorporation of multi-walled carbon nanotubes. When near-infrared light is shone upon the hydrogel, the photothermal effect of these nanotubes generates heat, causing the hydrogel to exhibit a stimulus response.

The overarching goal of this paper is to design and fabricate a bilayer-structured multifunctional hydrogel with simultaneously improved mechanical properties and controllable photothermal actuation. We hypothesize that (1) CNF reinforcement can effectively enhance the strength and ductility of PAM/SA hydrogels by forming a rigid bridging network; (2) the incorporation of MWCNTs and PNIPAM can endow the hydrogel with rapid near-infrared photothermal conversion and reversible thermo-responsive deformation; (3) the bilayer design (tensile layer and actuator layer) enables stable shape actuation under remote light stimulation. This paper aims to validate this hypothesis and provide a structural design strategy for hydrogel-based soft actuators.

## 2. Materials and Methods

### 2.1. Materials

The materials used in this paper include acrylamide (AM, 71.08 g/mol), PNIPAM (113.16 g/mol), sodium alginate (SA, 198.11 g/mol), cellulose nanofiber (CNF), N,N′-methylenebisacrylamide (MBAA, 99%, 154.17 g/mol), ammonium persulfate (APS, 98%, 228.2 g/mol), N,N,N′,N′-tetramethylethylenediamine (TEMED, 99%, 116.205 g/mol) and multiwall carbon nanotubes (MWCNTs). One of the multiwall carbon nanotubes is from Taixi Technology Co., Ltd. in Nanjing, China; and all the other materials are from Aladdin in Shanghai, China. 

Table 1 provides information on the composition of the hydrogel samples prepared in this paper.

### 2.2. Sample Preparation

Deionized water was used as the solvent, while AM and cross-linker MBAA were added sequentially with magnetic stirring (25 °C, 500 rpm, 1 h) until complete dissolution.

The preparation of the hydrogel precursor solution commenced by dissolving 4 g of AM in 40 milliliters of deionized water under magnetic stirring. Subsequently, 1 g of SA was added along with varying amounts of CNF (0 g, 0.1 g, 0.15 g, 0.2 g, 0.25 g, and 0.3 g, corresponding to mass fractions of 0%, 0.22%, 0.33%, 0.44%, 0.55%, and 0.66%, respectively) to prepare different CNF-reinforced hydrogel precursor solutions. The mixture was stirred for three hours until the CNF was uniformly distributed throughout the solution. Subsequently, 0.05 g of APS and 0.02 g of MBAA were added sequentially. After thorough mixing using a magnetic stirrer, we added 1 drop of TEMED. We continued stirring for 1 min and then poured the solution into the mold to allow cross-linking to take place. The cross-linking reaction was carried out at 25 °C for 2 h under ambient atmosphere to obtain hydrogels with different CNF contents for subsequent mechanical and structural characterization.

We added 1 g of sodium alginate to 40 g of deionized water in a water bath heated to 60 °C, stirring thoroughly for 1 h until fully dissolved. Subsequently, under magnetic stirring at 40 °C, we incorporated 4 g of PNIPAM and continued stirring for 1 h to form a homogeneous matrix solution. To achieve uniform dispersion of MWCNTs, 0.1 g of MWCNTs was first dispersed in 5 mL of deionized water containing 0.1 wt.% sodium dodecyl sulfate (SDS) via ultrasonic treatment for 60 min. The MWCNT dispersion was then gradually added to the PNIPAM/SA matrix solution, and the combined mixture was stirred magnetically for 3 h at 35 °C to ensure the homogeneous distribution of MWCNTs. At this stage, a PAM/SA hydrogel prepolymer solution modified with MWCNTs has been obtained. Thereafter, 0.05 g of APS and 0.02 g of MBAA were sequentially added, and we poured the solution into a custom bilayer mold. Cross-linking was conducted at 50 °C for 2 h to obtain the final photoresponsive hydrogel sample suitable for testing. The PAM/SA hydrogel co-modified with MWCNTs and CNF was prepared by adding CNF to the MWCNT-modified prepolymer.

### 2.3. Characterization

#### 2.3.1. Morphological Characterization

Scanning Electron Microscopy (Sigma300 of Carl Zeiss AG in Oberkochen, Germany) was utilized to examine the microstructural features of the hydrogels. Before testing, hydrogel samples underwent freeze drying for 48 h to remove water while preserving their internal porous structure. The dried samples were sputter-coated with a 10 nm layer of gold to improve electrical conductivity and avoid charging effects during imaging. SEM micrographs were obtained at an accelerating voltage of 5 kV, with magnifications ranging from 50× to 500×, to analyze the pore size distribution, uniformity, and dispersion of nanofillers (CNF and MWCNTs) within the PAM/SA matrix.

Image analysis was performed using the built-in SmartSEM software (V05.06 Carl Zeiss) to analyze the pore size distribution, structural uniformity, and dispersion of nanofillers within the PAM/SA matrix. For each formulation, three parallel samples were prepared and tested, and at least five different regions of each sample were observed to ensure the reproducibility and reliability of the morphological characterization.

#### 2.3.2. X-Ray Photoelectron Spectroscopy (XPS) Analysis

XPS measurements were conducted using an ESCALAB Xi+ X-ray photoelectron spectrometer (NexsaG2 of Thermo Fisher, Waltham, MA, USA) with an Al Kα X-ray source (hν = 1486.6 eV). Hydrogel samples were freeze-dried and ground into fine powders, which were then pressed into pellets for testing. Survey spectra were acquired over the binding energy range of 0–1200 eV to determine the elemental composition of the hydrogels. High-resolution spectra of C 1s, N 1s, and O 1s were recorded with a pass energy of 20 eV to analyze the chemical states and bonding configurations of these elements. Peak fitting was performed using Advantage software (Version 6.6.0) with Shirley background correction to resolve overlapping peaks and quantify the relative content of each chemical species.

#### 2.3.3. Thermosensitive and Photothermal Characterization

Photothermal conversion efficiency was characterized using a near-infrared (NIR) laser (DF-101S of Lichen Instrument Technology Co., Ltd. in Shanghai, China; wavelength: 808 nm, output power: 1.5 W) as the light source. Hydrogel samples were placed on a temperature-controlled stage, and an infrared thermal imager (Ti400, Fluke, Everett, WA, USA) was used to record the surface temperature of the samples in real time during NIR irradiation (0–300 s). The temperature–time curves were plotted to analyze the photothermal response rate and equilibrium temperature of the hydrogels. Additionally, the reversibility of the photothermal effect was verified by alternating NIR irradiation and natural cooling cycles.

In this paper, thermosensitive characterization refers to the temperature-dependent responsive behavior derived from the photothermal effect. The hydrogel’s thermosensitive response is reflected by the temperature elevation induced via NIR light excitation and the subsequent reversible deformation behavior under thermal stimulation. The photothermal effect and thermosensitive response are clearly distinguished; the former characterizes the energy conversion efficiency under NIR irradiation, while the latter represents the structural response of the hydrogel driven by the generated heat.

#### 2.3.4. Swelling Behavior Characterization

The swelling behavior of the hydrogels was investigated by immersing pre-weighed freeze-dried samples (*m*_0_) in deionized water at 25 °C. At predetermined time intervals (0, 10, 30, 60, 120, 180, 360 min), the samples were gently blotted with filter paper to remove the surface water and weighed (*m_t_*). The swelling ratio at each time point was calculated using the following formula:

$$SR(t)=\frac{m_{t}-m_{0}}{m_{0}}\times 100\%$$

The equilibrium swelling ratio (ESR) was determined when the mass change in the hydrogel was less than 1% over two consecutive measurements. Each sample was tested in triplicate, and the average values with standard deviations were reported.

### 2.4. Mechanical Characterization

The mechanical properties of the hydrogels were evaluated using a universal testing machine (Xiangjie, XJ810, Shanghai, China) equipped with a 1000 N load cell. The dimensions of the stretched samples were 50 mm × 10 mm × 1 mm (length, width and thickness, respectively). The strain was measured using a 30 mm gauge length, and the loading and unloading rates were set at 20 mm min^−1^.

## 3. Results and Discussion

### 3.1. Design and Characterization of CNF-Reinforced Hydrogels

During the process of reinforcing PAM/SA-based hydrogels with CNF, interfacial interactions within the hydrogel facilitate the uniform distribution of stress, thereby preventing sudden fracture of the hydrogel when subjected to stress. The reinforcing effect of the CNF significantly enhances the structural integrity of the hydrogel. It is worth noting that this design idea based on PAM/SA semi-IPN is essentially an important branch of composite network structure design.

In this composite hydrogel, in addition to the effects caused by the formed network structure itself, the amount of CNF used also influences the hydrogel’s properties.

A low content of CNF may not provide sufficient reinforcement, resulting in a hydrogel with low mechanical strength. On the other hand, a high content of CNF can lead to agglomeration, reducing the uniformity of the hydrogel and impairing its properties. Therefore, it is essential to determine the optimal CNF content for a specific application. In this paper, different contents of CNF were incorporated into the polyacrylamide matrix to investigate the effect of CNF content on the mechanical properties of the hydrogel.

**Figure 1:**
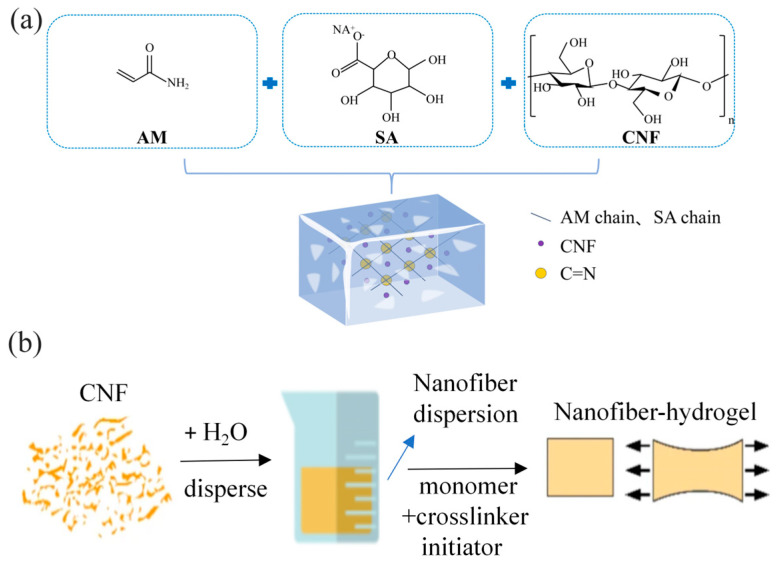
Fabrication of CNF-reinforced polyacrylamide hydrogel. (**a**) Reaction scheme and network formation. (**b**) Preparation flow chart.

**Figure 2:**
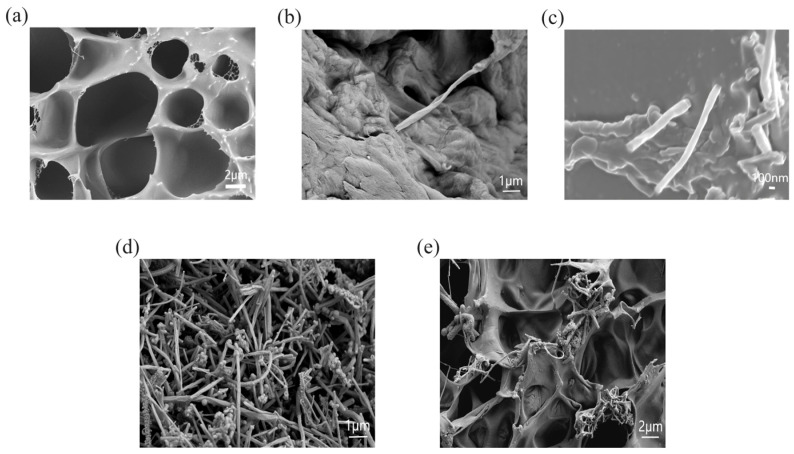
(**a**) SEM image of pure PAM hydrogel (scale bar, 2 μm). (**b**) SEM image of CNF (scale bar, 1 μm). (**c**) SEM image of CNF-reinforced PAM hydrogel (scale bar, 100 nm). (**d**) SEM image of MWCNTs (scale bar, 1 μm). (**e**) SEM image of MWCNT-reinforced PAM hydrogel (scale bar, 2 μm).

The fabrication strategy and microstructural characteristics of CNF-reinforced PAM hydrogels are depicted in Figure 1 and Figure 2, elucidating the interactions among components and the structural evolution during preparation.

Figure 1a depicts the molecular design of the hydrogel matrix: AM, SA, and CNF are integrated via free-radical polymerization (initiated by APS, cross-linked by MBAA) in deionized water. The structural formulas of AM, SA, and CNF highlight their hydrophilic functional groups (e.g., amide, hydroxyl, carboxylate), which enable hydrogen bonding and electrostatic interactions between components—laying the foundation for a robust composite network (schematized in the central box, where AM/SA chains, CNF, and cross-linking sites (C=N) are visualized).

Figure 1b outlines the stepwise preparation process of the CNF-reinforced hydrogel: CNF (as a supplementary filler) is first dispersed in water to form a stable suspension, which is then blended with the CNF dispersion, AM monomer, and crosslinker/initiator. In situ polymerization yields the CNF–hydrogel composite, whose enhanced mechanical ductility (schematically represented by the stretchable block) originates from the synergistic reinforcement of CNF.

The SEM micrographs in Figure 2 provide direct visual evidence of the structural evolution and reinforcement effects. Figure 2a reveals the porous architecture of the pristine PAM hydrogel, with interconnected pores of approximately 3–7 μm, which facilitate rapid water transport and ion diffusion. In contrast, Figure 2b shows the individual cellulose CNFs with a typical diameter of about 80 nm and high aspect ratio, which enable effective stress transfer within the hydrogel matrix. Figure 2c confirms the homogeneous dispersion of cellulose CNFs in the PAM matrix, where the CNFs bridge the pore walls and suppress crack propagation, leading to a denser and more robust network.

For completeness, Figure 2d,e also show the morphology of MWCNTs and their dispersion in the hydrogel matrix, which will be investigated in detail in the subsequent section on photothermal responsive hydrogels.

As shown in the SEM images of the semi-interpenetrating network hydrogel in Figure 2a, the pure PAM hydrogel exhibits a relatively loose and uneven porous structure. In contrast, the CNF-reinforced hydrogel shown in Figure 2c exhibits a relatively more uniform and compact porous structure in the observed field of view. It should be noted that the images Figure 2a,c were captured at different magnifications to highlight the respective microstructural features. The pure PAM hydrogel presents a relatively loose and uneven porous morphology within the respective observation scale, while the CNF-reinforced hydrogel shows denser pore walls and more homogeneous pore distribution, which preliminarily reflects the regulation effect of CNF on the hydrogel microstructure. This is attributed to the incorporation of CNF, which acts as a physical cross-linking point during polymerization. The CNF can restrict the random and excessive growth of PAM molecular chains and regulate the chain propagation behavior, thereby refining the pore structure. It should be noted that such regulation mainly affects the growth orientation and spatial distribution of PAM chains rather than significantly reducing the molecular weight of PAM. The uniform and compact porous structure of the CNF-reinforced hydrogel offers several advantages. First, it increases the specific surface area of the hydrogel, enhancing its adsorption capacity for water and other molecules. This is particularly important for applications such as drug delivery, where a large specific surface area allows for more efficient loading and release of drugs. Second, the small pore sizes prevent the leakage of encapsulated substances, such as drugs or enzymes, ensuring their stability and bioactivity. Third, the compact porous structure contributes to the hydrogel’s mechanical strength, as the evenly distributed pores enable it to better withstand external pressure.

**Figure 3:**
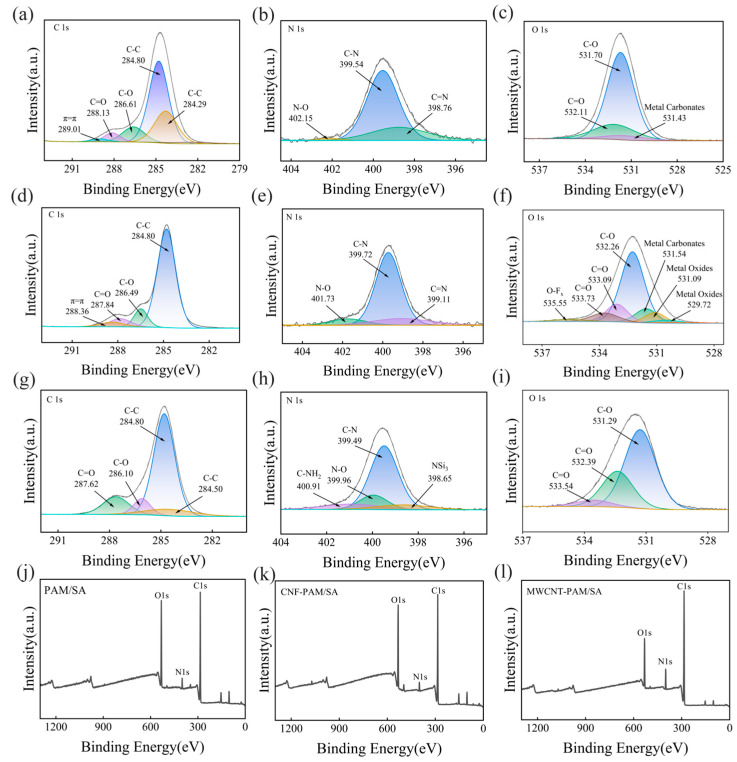
High-resolution XPS spectra of C 1s, N 1s, and O 1s for three acrylamide-based hydrogel systems. (**a**–**c**) The neat PAM hydrogel. (**d**–**f**) The CNF-reinforced PAM hydrogel. (**g**–**i**) The MWCNT-reinforced PAM hydrogel. (**j**) XPS full spectrum of PAM/SA hydrogel. (**k**) XPS full spectrum of CNF-PAM/SA hydrogel. (**l**) XPS full spectrum of MWCNT–PAM/SA hydrogel.

High-resolution XPS spectra (Figure 3) resolve the chemical states of C 1s, N 1s, and O 1s across three acrylamide-based hydrogel systems: neat PAM hydrogel (Figure 3a–c), cellulose-reinforced PAM hydrogel (Figure 3d–f), and MWCNT-reinforced PAM hydrogel (Figure 3g–i).

For the neat PAM hydrogel (Figure 3a–c), the C 1s spectrum features peaks at 284.80 eV (C-C/C-H, from the PAM backbone), 286.61 eV (C-O, amide groups), 288.13 eV (C=O, amide groups), and a faint π-π* satellite peak (289.01 eV, corresponding to PAM’s weak aromatic character). The N 1s spectrum consists of C-N (399.54 eV, amide bonds) and C=N (398.76 eV, amide resonance) plus a minor N-O signal (402.15 eV, trace oxidation). The O 1s spectrum is dominated by C-O (531.70–532.11 eV, amide oxygen) with a faint metal carbonate peak (attributed to substrate impurities).

In the CNF-reinforced PAM hydrogel (Figure 3d–f), the C 1s spectrum shows a prominent C-O peak (286.49 eV)—a signature of cellulose’s abundant hydroxyl/ether groups. The O 1s spectrum introduces O-F_x_ (535.55 eV, trace fluorine impurity) and metal oxides (529.72 eV, residual metals from cellulose) alongside shifted C-O peaks (533.09–533.73 eV, reflecting altered electronic environments of amide and cellulose hydroxyls). The N 1s spectrum retains C-N/C=N but with a higher C=N fraction (399.11 eV), suggesting cellulose-induced structural changes to PAM’s amide groups.

For the MWCNT-reinforced PAM hydrogel (Figure 3g–i), the C 1s spectrum displays dual C-C peaks: 284.80 eV (PAM backbone) and 284.50 eV (MWCNT sp^2^ carbon) with a reduced C=O fraction—consistent with MWCNT’s sp^2^ carbon dominance. The N 1s spectrum includes new signals for C-NH_2_ (399.96 eV, from amine-functionalized MWCNTs) and N-Si (398.65 eV, residues of MWCNT dispersants). Notably, the O 1s spectrum lacks metal oxides (MWCNTs contain minimal metal impurities) and shows C-O peaks (531.29 eV) comparable to neat PAM.

Together, these results confirm that CNF and MWCNTs impart distinct chemical signatures to the hydrogel matrix, which are aligned with their unique structural roles. Beyond confirming the successful incorporation of cellulose and MWCNTs into the PAM network, the XPS data provide critical insights into the interfacial interactions between the PAM matrix and reinforcing phases: the enhanced C-O content in the cellulose-reinforced hydrogel indicates the formation of hydrogen bonds between cellulose hydroxyl and PAM amide groups, which is anticipated to improve the mechanical strength of the composite. For the MWCNT-reinforced hydrogel, the presence of amine-functionalized groups (C-NH_2_) suggests potential covalent or non-covalent interactions between MWCNTs and the PAM matrix, laying the foundation for enhanced mechanical performance. These findings not only validate the structural design of the hydrogel composites but also provide a theoretical basis for optimizing their performance in targeted applications.

### 3.2. Mechanical Properties of Nanofiber-Reinforced Hydrogels

The mechanical properties of the CNF-reinforced hydrogels are significantly influenced by the content of CNF and the mass ratio of PAM to SA, as shown in Table 2.

It can be seen from Table 2 that the mechanical properties of the CNF-reinforced hydrogels are significantly affected by the CNF content and the mass ratio of PAM to SA. With the increase in SA content from 0 wt.% to 20 wt.% (PAM:SA from 1:0 to 4:1), the tensile strength and elongation at break of the hydrogel have been significantly enhanced. This is because SA introduces additional ionic cross-linking points, which enhance the interaction between polymer chains and improve the flexibility of the network. However, when the SA content further increases to 50 wt.% (PAM:SA = 1:1), the tensile strength decreases to 3 kPa, as excessive SA leads to phase separation between PAM and SA, weakening the network structure.

For the CNF-reinforced hydrogels (PAM:SA = 4:1), the tensile strength and elastic modulus first increase and then decrease with the increase in CNF content. When the CNF content is 0.44 wt.%, the tensile strength reaches the maximum value of 17 kPa (89% higher than pure PAM). This is due to the uniform dispersion of CNF within the hydrogel matrix, which forms a rigid reinforcement network and effectively transfers external loads. However, when the CNF content exceeds 0.44 wt.%, agglomeration occurs, resulting in stress concentration points within the hydrogel and thus a decrease in mechanical properties.

The mechanical property values presented in Table 2 are comparable to those of soft hydrogel actuators and flexible sensing materials reported in recent studies, highlighting their practical applicability in targeted scenarios. For instance, Sang et al. [38] developed CNF-mediated conductive hydrogels for wearable sensors, which exhibited a tensile strength of ~8.5 kPa and elongation at break of ~580%, while our optimized PAM/SA (4:1) hydrogel with 0.44 wt.% CNF achieves a higher tensile strength (17 kPa) and significantly improved ductility (900% elongation). Similarly, Lei et al. [13] reported PNIPAM-based temperature-responsive ionic conductive hydrogels with tensile strengths of 8–12 kPa and elongation of 450–650% for flexible sensing applications, whereas our CNF-reinforced hydrogel surpasses these performance metrics, particularly in terms of fracture strain. Even in comparison to hydrogels designed for soft actuation, such as the AMF-controlled magnetothermal hybrid hydrogel fiber mats by Shen et al. [37] (tensile strength ~14 kPa, elongation ~750%), our material maintains competitive mechanical performance while integrating photothermal responsiveness. This superiority stems from the synergistic reinforcement of CNF and SA: the semi-interpenetrating network (semi-IPN) formed by PAM and SA provides a flexible matrix, while uniformly dispersed CNF acts as rigid cross-linking points to dissipate stress, avoiding localized fracture [19,38]. Thus, despite the absolute values of tensile strength being lower than those of structural hydrogels, the balanced strength–ductility profile of our CNF-reinforced hydrogel is well suited for soft actuators that require both mechanical robustness and deformability.

**Figure 4:**
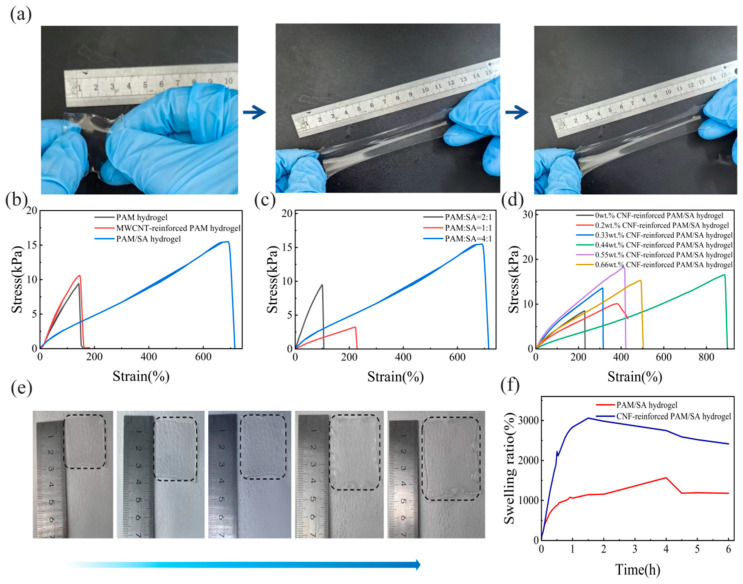
Mechanical performance and swelling behavior of CNF-reinforced polyacrylamide hydrogels. (**a**) Macroscopic tensile deformation process of the prepared hydrogel. (**b**) Stress–strain curves of pure PAM hydrogel, MWCNT-doped PAM hydrogel, and sodium alginate hydrogel. (**c**) Stress–strain curves of hydrogels with different PAM/SA mass ratios. (**d**) Stress–strain curves of hydrogels reinforced with varying CNF contents. (**e**) Visual swelling state evolution of the hydrogel over time. (**f**) Swelling ratio–time profiles of pure PAM hydrogel and fiber-reinforced hydrogel.

The mechanical performance (tensile behavior) and swelling characteristics of CNF-reinforced PAM hydrogel were systematically evaluated, as presented in Figure 4.

Figure 4a illustrates the macroscopic tensile deformability of the as-fabricated CNF-reinforced hydrogel. The sample can be elongated to several times its original length without fracture, verifying its exceptional ductility, which is a critical characteristic facilitated by the CNF-reinforced network architecture.

Figure 4b compares the stress–strain responses of three hydrogel systems: pristine PAM hydrogel, MWCNT-doped PAM hydrogel, and SA hydrogel. The pristine PAM hydrogel exhibits low tensile strength and limited strain (failing at about 130% strain), while the MWCNT-doped PAM hydrogel shows marginally improved strength but remains moderately ductile. In contrast, the SA hydrogel displays substantially higher tensile strength and elongation, highlighting the reinforcing effect of SA on the hydrogel matrix.

Figure 4c further investigates the impact of PAM/SA mass ratios (PAM:SA = 2:1, PAM:SA = 1:1, PAM:SA = 4:1) on mechanical performance. Among these formulations, the PAM:SA = 4:1 hydrogel (higher SA content) demonstrates the highest tensile strength and ductility, whereas the PAM:SA = 2:1 hydrogel exhibits the lowest mechanical performance. This trend confirms that an increase in SA content improves the hydrogel’s network toughness by providing additional cross-linking sites.

Figure 4d presents the stress–strain curves of hydrogels modified with varying CNF contents (0–0.66 wt.%), which clarifies the effect of CNF doping on the mechanical performance of the CNF-reinforced matrix. For the 0 wt.% CNF (control hydrogel sample) (black curve), stress increases gradually with strain but exhibits limited ductility, failing at a relatively low strain. In contrast, all CNF-doped hydrogels show enhanced tensile strength and elongation: as the CNF content rises (from 0.20 to 0.66 wt.%), both the maximum stress and fracture strain of the hydrogels increase significantly. For example, the 0.55 wt.% CNF-doped hydrogel (purple curve) achieves a peak stress of about 18 kPa at a strain of 400%, while the 0.44 wt.% CNF-doped hydrogel (green curve) exhibits the highest ductility with a fracture strain approaching 900% (and a corresponding stress of 17 kPa). This trend confirms that CNF act as effective reinforcing fillers: their uniform dispersion within the hydrogel matrix creates a robust network that dissipates mechanical energy during deformation (via CNF–matrix interfacial interactions and crack bridging), thus improving both the strength and ductility of the hydrogel. Notably, the 0.44–0.55 wt.% CNF range balances these two properties optimally, while excessive CNF content (0.66 wt.%) may lead to partial agglomeration, slightly reducing ductility (though strength remains elevated).

In addition to assessing tensile properties, the swelling behavior of the hydrogel was examined (Figure 4e–f). Figure 4e illustrates the visual swelling process of the hydrogel over 6 h: the sample gradually expands and becomes more translucent, which is consistent with water absorption by the hydrophilic network. Figure 4f compares the swelling ratio–time profiles of the pristine PAM hydrogel and fiber-reinforced hydrogel: the pristine hydrogel undergoes rapid initial swelling (reaching about 3000% within 1 h) followed by a slight decrease, while the fiber-reinforced hydrogel exhibits a slower, more stable swelling process (equilibrium swelling ratio about 1500% at 6 h). The reduced swelling ratio of the reinforced hydrogel is attributed to the CNF network, which limits excessive expansion of the PAM matrix and enhances its mechanical stability.

Collectively, the integration of SA and CNF (along with tailored MWCNTs doping) synergistically enhances the tensile ductility, strength, and swelling controllability of PAM hydrogels, providing a tunable framework for engineering robust hydrogel materials.

### 3.3. Thermosensitivity of Nanofiber-Reinforced MWCNT-Doped Photosensitive Hydrogels

The incorporation of responsive functional groups represents a fundamental strategy for constructing multi-stimulus-responsive hydrogels. Grafting temperature-sensitive PNIPAM onto the PAM backbone enables the hydrogel to respond to temperature changes with the mass ratio of PNIPAM to PAM being critical in modulating the thermosensitive responsiveness of the hydrogel matrix. Specifically, the thermosensitive performance of the hydrogel exhibits a positive correlation with the PNIPAM content in the matrix (composed of PNIPAM and PAM), where a higher proportion of PNIPAM relative to PAM is advantageous for achieving enhanced thermosensitive response. In this paper, the mass ratio of PNIPAM to PAM was maintained within the range of 4:1. Experimental results indicated that the thermosensitive responsiveness of the hydrogel significantly improved as the proportion of PNIPAM in the matrix increased. This enhancement is attributed to the increased PNIPAM content, which provides a greater number of temperature-sensitive segments within the hydrogel network, enabling more rapid and pronounced conformational changes in response to temperature fluctuations.

By combining CNF surface functionalization, the interfacial interactions between CNFs and polymers can be further enhanced. When the environmental temperature changes, the CNF–polymer interface interactions dynamically adjust, thereby driving the hydrogel to undergo volume phase transitions or shape memory effects. Upon temperature elevation, PNIPAM-grafted polyacrylamide hydrogels undergo volumetric contraction with CNFs enhancing the stability and reversibility of this response. This approach enables the fabrication of smart hydrogel materials exhibiting diverse stimulus-responsive properties, catering to diverse application requirements.

The thermosensitive responsiveness and photothermal-driven deformation behavior of the CNF-reinforced hydrogel were systematically characterized under near-infrared (NIR) light irradiation.

**Figure 5:**
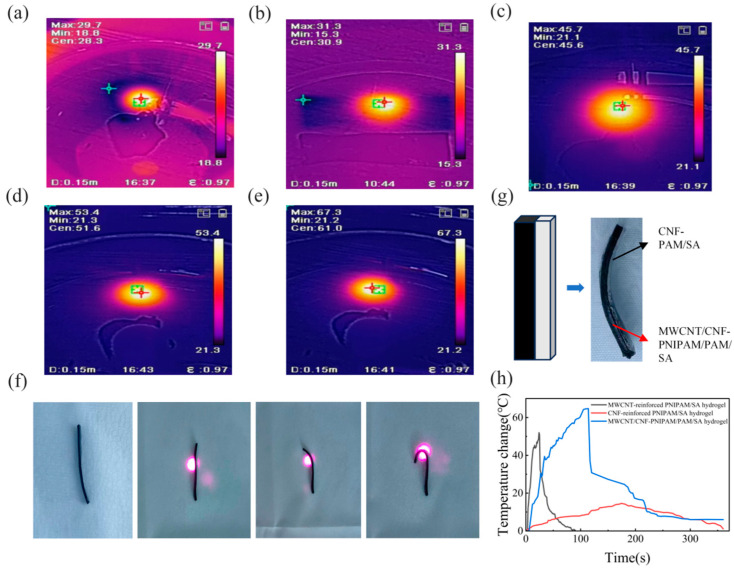
Photothermal response and thermally induced deformation of CNF-reinforced MWCNT-doped photosensitive hydrogels under NIR irradiation. (**a**–**e**) Infrared thermal images of the hydrogel at different irradiation time points. In each thermal image, the red cross symbol marks the position of the maximum temperature, while the green cross symbol marks the position of the minimum temperature. (**f**) Time-dependent bending deformation behavior of the hydrogel. (**g**) Hydrogel-based dual-layer infrared-driven structure. (**h**) Temperature–time curves of three hydrogel groups with different compositions.

Figure 5a–e present the infrared thermal images of the hydrogel at different irradiation durations, which were captured by an infrared thermometer. It should be noted that the CNF reinforcement in the hydrogel system mainly consists of MWCNTs, which serve as the core photothermal conversion medium. When exposed to NIR irradiation, MWCNTs efficiently absorb NIR photons and convert the light energy into thermal energy through the photothermal effect, which is a process that is rapid and highly efficient. The generated heat is then quickly transferred to the entire hydrogel matrix, leading to a significant increase in the hydrogel’s temperature. The maximum temperature (Max) of the hydrogel increased from 29.7 °C (initial state, Figure 5a) to 67.3 °C (Figure 5e) with prolonged NIR irradiation, while the ambient temperature remained stable (≈21 °C). This rapid temperature rise further triggers the deformation of the hydrogel; such deformation is typically attributed to the temperature-sensitive nature of the hydrogel matrix. This result confirms the efficient photothermal conversion capability of the CNF-reinforced hydrogel, which can rapidly accumulate heat upon NIR stimulation.

Corresponding to the temperature rise, the hydrogel exhibited reversible time-dependent bending deformation (Figure 5f). The initially straight hydrogel gradually curled into a “C” shape as the irradiation time increased, and it rapidly recovered its initial straight shape within a short period after removing the NIR light in a room-temperature water bath. This shape evolution originates from the asymmetric swelling/deswelling behavior induced by photothermal heating and the anisotropic reinforcement of CNFs. The fast and complete shape recovery confirms the reversible thermosensitive response of the bilayer hydrogel rather than irreversible deformation. Figure 5g displays the actual experimental setup and the hydrogel sample in the testing process, verifying the feasibility of the in situ photothermal driving test.

To quantify the thermosensitive performance, Figure 5h compares the temperature–time curves of three well-defined hydrogel groups: the target CNF-reinforced MWCNT-doped PNIPAM/PAM/SA bilayer hydrogel, the control CNF-reinforced PNIPAM/PAM/SA hydrogel without MWCNT doping, and the control MWCNT-doped PAM/SA hydrogel without CNF and PNIPAM. The target bilayer hydrogel displayed the fastest temperature rise, reaching approximately 70 °C within 100 s and retaining the highest equilibrium temperature. In comparison, the CNF-only reinforced hydrogel showed only a minor temperature increase (≤10 °C), and the MWCNT-only doped hydrogel exhibited poor thermal stability. These results verify that the synergistic combination of MWCNT-enabled photothermal conversion, CNF-mediated mechanical reinforcement, and PNIPAM-based thermosensitivity grants the hydrogel excellent photothermal responsive performance.

In summary, the CNF-reinforced hydrogel integrates efficient photothermal conversion and stimuli-responsive deformation, which originates from the synergistic effect of CNF-mediated photothermal absorption and the thermosensitive volume change in the polyacrylamide matrix. This thermosensitive responsiveness endows the hydrogel with potential for remote-controlled soft actuator applications.

## 4. Conclusions

This paper systematically explores the design, preparation, and performance optimization of CNF-reinforced PAM hydrogel to overcome the limitations of conventional single-function hydrogels. In this paper, two types of hydrogels were designed and prepared: one was a CNF-reinforced hydrogel with improved mechanical properties, and the other was a photothermal-responsive hydrogel incorporating MWCNTs and PNIPAM. Through the design of a bilayer structure, a multifunctional hydrogel with enhanced mechanical properties and thermosensitive responsiveness was developed.

The optimized hydrogel (PAM/SA ratio of 4:1, CNF content of 0.44 wt.%) exhibited a tensile strength of 17 kPa (89% higher than that of pure PAM) and an elongation at break of up to 900%. These properties surpass those of most CNF-reinforced hydrogels and PNIPAM-based thermosensitive hydrogels reported in recent studies. Under near-infrared irradiation, the hydrogel can heat up to 60 °C within 100 s and undergo reversible bending deformation, fully recovering in a room-temperature water bath without the need for light stimulation. Compared with similar photothermal hydrogel systems, this material achieves a better balance between the mechanical strength, photothermal conversion efficiency and reversibility of the driving mechanism.

Mechanical optimization is attained through the synergistic regulation of the PAM/SA ratio and nanofiller content, which enhances the semi-interpenetrating network structure via interfacial interactions including hydrogen bonding. This approach effectively balances mechanical strength and ductility while improving swelling stability. The incorporation of PNIPAM and MWCNT provides the hydrogel with efficient near-infrared-triggered photothermal conversion and reversible bending actuation, which are mechanisms that are substantiated by XPS-verified chemical bonding and interactions among functional groups.

This paper presents a novel fabrication strategy for multifunctional hydrogels, thereby broadening their applications in motion tracking, drug delivery, and smart sensing. It establishes a foundation for next-generation hydrogel-based systems with future research aimed at enhancing biological stability, multi-stimulus responsiveness, and industrial scalability.

## Figures and Tables

**Table 1:** Designation, formulation and chemical composition of all hydrogel samples in this paper.

Sample ID	Sample Name	PAM:SA Mass Ratio	CNF Content (wt.%)	PNIPAM	MWCNT
1	Pure PAM hydrogel	1:0	0	-	-
2	PAM/SA-4:1	4:1	0	-	-
3	PAM/SA-2:1	2:1	0	-	-
4	PAM/SA-1:1	1:1	0	-	-
5	PAM/SA-4:1-NC0.2	4:1	0.22	-	-
6	PAM/SA-4:1-NC0.33	4:1	0.33	-	-
7	PAM/SA-4:1-NC0.44	4:1	0.44	-	-
8	PAM/SA-4:1-NC0.55	4:1	0.55	-	-
9	PAM/SA-4:1-NC0.66	4:1	0.66	-	-
10	CNF-reinforced PNIPAM/SA	4:1	0.44	+	-
11	MWCNT-reinforced PNIPAM/SA	4:1	0	+	+
12	MWCNT/CNF-PNIPAM/PAM/SA	4:1	0.44	+	+

Note: “+” = component incorporated; “-“ = component not incorporated.

**Table 2:** Mechanical properties data for CNF-reinforced hydrogels.

Sample	PAM:SA Mass Ratio	CNF Content (wt.%)	Tensile Strength (kPa)	Elongation at Break (%)	Elastic Modulus (kPa)
Pure PAM	1:0	0	9	200	0.09
PAM/SA-4:1	4:1	0	16	700	0.023
PAM/SA-2:1	2:1	0	9.5	200	0.02
PAM/SA-1:1	1:1	0	3	200	0.02
PAM/SA-4:1 -NC0.2	4:1	0.2	10	200	0.1
PAM/SA-4:1 -NC0.33	4:1	0.33	13.5	400	0.068
PAM/SA-4:1 -NC0.44	4:1	0.44	17	900	0.019
PAM/SA-4:1 -NC0.55	4:1	0.55	18	400	0.09
PAM/SA-4:1 -NC0.66	4:1	0.66	15.5	500	0.078

## Data Availability

The original contributions presented in this paper are included in the article material. Further inquiries can be directed to the author.
